# Exploring Students’ Emotional Well-Being in the Ideal University Hostel Using the Qualitative Repertory Grid Technique

**DOI:** 10.3390/ijerph20186724

**Published:** 2023-09-07

**Authors:** Fanan Jameel, Ahmed Agiel

**Affiliations:** Department of Architectural Engineering, United Arab Emirates University, Abu Dhabi P.O. Box 15551, United Arab Emirates; 201570349@uaeu.ac.ae

**Keywords:** emotional well-being, ideal university hostel, personal construct theory, qualitative repertory grid technique, students’ perceptions

## Abstract

One of the ramifications of the COVID-19 pandemic is that it has lent urgency to ongoing discussions on mental well-being, particularly among university students. While standard techniques are available to diagnose mental health disorders such as depression, anxiety, and stress, ambiguity persists regarding the emotional aspect of well-being. Emotional well-being (EWB) is a recently developed concept that seeks to understand the contribution of emotions to one’s well-being. Interactive approaches for such investigations are recommended to understand people’s contextual experiences in the built environment. This study utilizes a qualitative approach, underpinned by personal construct theory (PCT) and the qualitative repertory grid technique (RGT), to understand how university hostel designs can contribute to students’ emotional well-being. We interviewed fifteen students from the United Arab Emirates University (UAEU) and obtained their perceptions of three built environments they experienced and an ideal place they imagined. The results unveiled design-related factors associated with students’ emotional constructs and elucidated characteristics of an ‘ideal’ hostel in response to these emotional constructs. These findings enrich our knowledge of EWB within university hostels offering insights for the future design that consider the emotional aspect of well-being for residents.

## 1. Introduction

Mental health is one of the three aspects of well-being. The World Health Organization (WHO) defines it as “a state of well-being in which an individual realizes his or her own abilities, can cope with the normal stresses of life, can work productively, and is able to make a contribution to his or her community” [1]. This definition is vague in its reference to one’s emotional states, and considerable effort has been dedicated to explaining the influence of emotions on mental well-being [2,3]. Emotional well-being (EWB) is a recently developed concept that serves as an umbrella for the different terms used to refer to the psychological aspects of well-being. It involves understanding the contributions of emotions to well-being [4]. There are three main theories of emotions that differ in their causes: the physio-logical theory based on responses within the human body [5], the neurological theory based on responses within the human brain [6], and the cognitive theory based on the subject’s thoughts [7]. The correlation between the built environment and emotions has been objectively considered in architecture, where different biometric tools are used to measure certain emotions [8,9,10]. On the contrary, subjectivity has rarely been used to identify people’s emotional needs based on the cognitive theory of emotions. Since the instant emotional response of users to certain stimuli within the built environment does not guarantee clarity regarding their long-term preferences, there is a need to understand people’s emotions by using qualitative inter-active approaches based on psychological methods [11]. 

Issues related to the mental aspect of well-being, especially among young people and females, have attracted widespread interest since the outbreak of the COVID-19 pandemic. For example, the global prevalence of anxiety and depression increased by 25% in the first year of the pandemic [12]. A number of researchers in Bulgaria, China, France, Germany, Italy, Turkey, India, and the United Arab Emirates have investigated personal well-being during this time, with a focus on mental well-being [13,14,15,16,17]. The general aim is to quantitatively measure certain pre-defined mental health disorders. Cross-sectional studies have been conducted on university students enrolled in various academic programs. Standardized psychiatric scales, such as the Patient Health Questionnaire (PHQ-9) to measure depression and the General Anxiety Disorder (GAD-7) to measure anxiety, have been the main tools used to this end based on online self-reporting questionnaires. The findings common to these studies pertain to significant levels of mental health disorders among students, including depression, stress, and anxiety. Some studies also found that females reported higher levels of such disorders, as did students residing in hostels on campus [13,17]. However, few studies have sought to correlate the levels of mental health disorders with the design of the built environments in which the students reside [13,15,18]. The correlations identified in the literature included common attributes of the physical design, such as proximity to nature and factors influencing the indoor environmental quality (IEQ).

The COVID-19 pandemic provided an opportunity to learn new lessons about the impact of buildings on people’s well-being that otherwise might have remained undiscovered [19]. Studying well-being during the pandemic was more conducive to a qualitative method of investigation rather than a quantitative method of measuring. One of the few studies that applied a qualitative descriptive basis was conducted in China, in which the authors sought to record the participants’ experiences in quarantine by using phone interviews [20]. Their findings highlighted several feelings that participants had in common in various stages of quarantine, such as feeling afraid, impatient, nervous, and calm. The study also identified the importance of feeling optimistic as a coping strategy and feeling comfortable with one’s living environment as an external support. We identified a link between such descriptions of people’s perceptions and the design of the built environment in a pilot study [21]. Perceptions of quarantined and non-quarantined students who stayed in the same hostel of Maqam 4, a hostel for female students at the United Arab Emirates University (UAEU), were gained through semi-structured individual interviews. They mentioned common positive feelings of comfort and satisfaction and linked them to some design-related factors, such as the availability of sufficient natural daylight, the suitability of artificial light, the good quality of views from windows, and the new and colorful look of buildings. On the other hand, the bedroom size, floor layout, and allocation of the hostel negatively affected the stay experience of quarantined perceivers. 

The lessons learned from COVID-19 include insights into the types of buildings that can promote personal well-being. Two recent reviews have claimed that the concept of well-being in a built environment shifts from one of mitigating the negative by following certain design principles and strategies to that of accentuating the positive by designing structures to promote experiences in which the emotional aspect is evident. More interactive approaches to such investigations are required to enrich our knowledge of people’s subjective well-being (SWB) by considering boundaries of their preferences in relation to contextual scenarios [22,23]. Considering that female university students are among the demographic most vulnerable to issues concerning mental well-being, this study investigates their experiences by using an interactive approach to gain insights into their perceptions of the emotional impact of the built environment as well as their views on the ideal university hostel. The work here enriches our understanding of EWB in the built environment and can orient the future designs towards emotionally healthy buildings.

## 2. Methods

We used the above-mentioned pilot study as the basis and extended it after the outbreak of COVID-19 to incorporate a larger number of students. The qualitative results reported in this paper were obtained by implementing concepts of the personal construct theory (PCT) and the qualitative repertory grid technique (RGT). The validity of using the RGT in the built environment was recently assessed in a review, and the results verified its potential for use in this context [24]. 

### 2.1. Repertory Grid Technique

The RGT is an interview technique that follows a structured format in which the interviewee’s responses are arranged in a grid-like structure. It was created by George Kelly in 1955 and is based on his PCT [25]. The RGT consists of elements, which are the objects or topics being investigated, constructs, which represent people’s perceptions of these elements, and a rating grid. There are different forms of the RGT, and the choice of the form depends on the specific focus of the research. Quantitatively, the RGT can be implemented in any of four forms: real repertory grids, grids with fixed elements, grids with fixed constructs, and grids with both fixed elements and constructs [26]. These forms all use a quantitative approach, whereby the interviewee rates each element by using a Likert scale based on each construct. However, there is also a qualitative form of the RGT in which people’s perceptions, along with their constructs, are recorded by using their own words as responses to the elements being studied [27]. 

The contents of people’s thoughts, which they connect with their ideas or principles, are used as the elements here. These elements possess certain shared features. They need to be consistent, of the same type, separate and inclusive, and to cover a significant portion of the topic under examination. They also need to be concise, definite, effortlessly comprehensible to the participant, and, ultimately, familiar to the interviewee through their experiences [28].

The researcher can either provide the elements to the participants or ask them to generate them. If the researcher provides the elements, their number depends on the subject matter being investigated and the allotted interview time. Alternatively, to elicit the elements, the researcher can use direct or indirect questions to enable the participants to identify what they consider to be the most important elements of the topic under investigation. This method enables the respondents to nominate the crucial elements that constitute the subject being studied [29].

The constructs represent the distinctions that individuals use to describe the elements in their own words. A suitable construct should be a two-part phrase that contains opposite terms. This polarity is determined by gathering similarity and difference statements from the interviewees. Jankowicz claimed that a good construct should be clear, sufficiently detailed, and pertinent to the topic under investigation [30]. The approaches used to elicit constructs significantly vary depending on the nature of the research and the topic under investigation. The conventional method of eliciting constructs involves comparing the elements in a monadic, dyadic, or triadic form [26]. However, constructs can also be elicited through means other than directly examining the elements. For instance, personal constructs can be elicited by conducting interviews that involve discussing the elements in detail. This approach makes the interview more engaging and conversational for the interviewee, as opposed to being a tedious task [27].

### 2.2. Research Design

The main aim in this study was to understand how the built environment influences people’s emotional well-being, and accordingly, design the ideal place where their emotional needs are considered. To conduct the study, female university students were considered as the participants to obtain their perceptions about the types of built environments they experienced. The significance of choosing the category of female university students refers to their high vulnerability to issues concerning mental well-being [13,17]. Therefore, this study will investigate female university students’ emotional well-being in different built environments they experienced and construct the characteristics of an ideal university hostel where the students’ emotional needs are promoted. The university students’ hostel is not just a dwelling; it serves as an educational hub. Besides, it can foster personality development, as students also acquire essential skills to nurture their individual talents and embrace independent living [31]. It can be available in different forms worldwide [32]. Therefore, considering the students’ hostel as a multi-functional place, this study can help to understand how other typologies of buildings can support residents’ emotional well-being.

As the PCT focuses on people’s perceptions based on their experiences of the same event, place, or object [25], it was important to consider a sample of students who shared common demographic characteristics and had lived in the same hostels. Undergraduate Emirati female students at the UAEU were targeted for this study, and ethics approval was obtained from the relevant committee of the university. We first interviewed a student housing specialist who provided a list of students who had lived in their homes as well as two types of hostels on the UAEU campus. The students lived in their homes during their first academic semester and took online courses. They then lived in the Maqam 4 hostel the next semester. Once they had completed their first academic year, they were moved to the New Campus (NC) hostel. They thus spent almost one academic semester in each of their homes, the Maqam 4 hostel, and the NC hostel. The importance of having more than one built environment that the students had experienced is based on the idea of comparison, which constitutes the core of the PCT. This concept suggests that people can better describe their perceptions of an element when they compare it with others [25]. Therefore, the three experienced built environments for each student, her home, Maqam 4, and the New Campus, became the three elements of discussion to elicit students’ constructs grounded in the substance of the PCT. The two hostels of the UAEU had been recently built within the last ten years following international standards and thus were suitable for consideration in this study. To have a holistic view about the design of the two hostels, architectural drawings, obtained from the Department of Campus Development of the UAEU, and photos taken by the researcher are attached in Appendix A. While the homes of students vary from one to another, they still share common characteristics. Usually, Emiratis families are big with more than five members and extended families living in privately owned villas within a residential neighborhood. The villas have spacious indoor spaces and private outdoor greenery spaces, and a private bedroom and bathroom per female student and/or shared with a sister. These characteristics were commonly mentioned by the participants of the study and can been seen in an example of a typical Emirati villa designed by the researcher for one Emirati family in Appendix B. It is important to mention that the primary focus was not on determining which setting provides a better emotional experience for students. Instead, the main objective was to gain insight into what factors contribute to a positive emotional experience in a living environment. By examining the students’ emotional constructs and their associations with design-related factors, the study aimed to identify what elements contribute to fostering a positive and enriching living experience for individuals.

### 2.3. Sampling

The RGT is known for its intensive nature, which typically leads to smaller sample sizes. However, with the advent of new repertory grid systems, larger sample sizes can also be accommodated. This allows for the generation of quantitative data, which is useful for understanding trends and patterns within a larger population. On the other hand, when the focus is on qualitative data, a sample size of 15–25 individuals within a specific population is often adequate. Even with this relatively small sample size, researchers can frequently derive enough essential concepts to capture and approximate the “universe of meaning” related to a particular area of discussion or domain of discourse. This means that despite the limited number of participants, the study can still provide valuable insights and understanding into the subject matter [33,34]. Additionally, the insights gained from the RGT with a small sample can be utilized to develop items for larger sample instruments. For instance, researchers can use the information gathered from the initial qualitative study to create closed-ended and pre-prepared constructs, which can be administered to a larger sample for further quantitative analysis. This approach allows researchers to combine the strengths of qualitative and quantitative methods for a more comprehensive understanding of the subject under investigation [24].

Given the nature of this study, we used purposive sampling to interview the representative students. We created an online survey through Google Forms and sent it to 390 students. We obtained a list of 22 students who could have been interviewed for this study. We subsequently contacted them and conducted 15 one-on-one semi-structured interviews. The interviewees were all around 19 years old, were enrolled in different colleges/programs, and were from different Emirates.

### 2.4. Procedure

Considering the focus of this study, we used the qualitative form of the RGT here, that was used in the pilot study, and the results revealed the potential of using the RGT in a full-scale study [21]. Individual semi-structured interviews were conducted to this end. The elements of discussion were the three places that students experienced. We asked the following set of pre-determined questions in each interview:

1. When comparing the three places, how do you describe your living experience in each?
2. When comparing the three places, where did you emotionally feel the best, and why?
3. What are the design-related aspects of these spaces that you feel influenced you positively?
4. What are the design-related aspects of these spaces that you feel influenced you negatively?
5. If you imagine an ideal place for university students, what would it be like?

To ensure the focus of the interview on the design of the built environments and to help students reflect on their experiences, they were encouraged to think of the questions by comparing the three experienced built environments: homes and the two hostels. Props were continually provided to explore the meanings of the expressed thoughts. The interview took on a conversational tone in a quiet environment with minimal inference by the interviewer, allowing students to freely articulate their feelings. The 15 interviews were individually conducted by telephone, and each lasted approximately 30 min. Interviews were digitally recorded using a handheld recorder with the permission of the participants. Although data saturation was achieved at the 10th interview, when it became clear that there were no new themes, the other 5 participants were interviewed to emphasize commonality.

### 2.5. Data Processing and Analysis

The interviews were transcribed and translated into English. The transcripts were then thematically analyzed [35] as this is efficient for analyzing qualitative data generated based on the PCT [36,37,38]. The data were coded based on their distinguished meanings and commonality in wording [39] and were then categorized into themes featuring internal homogeneity and external heterogeneity [40]. These themes were derived from the data, rather than from prevalent theoretical or conceptual frameworks. The transcripts were read and reread to develop the codes. A multi-coding approach was used, with frequent discussions with the co-researcher to refine the themes until a consensus had been reached on the final themes.

## 3. Results

The results were categorized into three main themes through thematic analysis. These themes were substantiated by several subthemes that exemplified the participants’ perceptions. The first theme was obtained from the first question in the interview, regarding the students’ general perceptions of their living experiences. This introductory part was important not only to capture the content of a living experience for students, but also to help the interviewees adapt to the nature of study. The second and main theme of the results, gained from the second, third, and fourth questions, represented the students’ perceptions of their emotional well-being, which was identified as one of the aspects of their perceptions that formulated their living experiences. The last theme, gleaned from responses to the fifth question, reflected the students’ perceptions of an ideal place to live, where their emotional well-being was adequately supported.

In each of the three main themes described in the following sections, there are various sub-themes that have emerged. These themes and sub-themes represent the patterns and categories that emerged from the analysis of the data collected during the interviews. Within these sub-themes, students expressed specific emotional constructs that were linked to various design-related factors. Some students conveyed their emotions more effectively when comparing their experiences living at home versus the hostels, while others found it easier to describe their feelings by comparing the two hostels. It is essential to note that the purpose of these findings was not to determine which living environment, whether it be home or a hostel, offers a better emotional experience for students. Instead, the primary objective was to gain a deeper understanding of the aspects that contribute to a positive emotional experience in a living environment. This insight can be valuable for architects, designers, and planners in creating more emotionally supportive and conducive living spaces for students and potentially other individuals in various settings.

### 3.1. Students’ Perceptions of Their Living Experiences

The students’ living experiences mainly revolved around four dimensions: the level of concentration when studying, sleeping behaviors, the capability of accessing the means of catering to their daily needs, and their emotional status. By comparing the three built environments—their home, the Maqam 4 hostel, and the NC hostel—the students described the impacts of different design-related factors on these dimensions. The presence/absence of sound insulation, which determined the extent of noise, was mentioned the most often by the respondents. For example, they claimed that its absence in the NC hostel disrupted their focus while studying, led to trouble sleeping, and affected their privacy.

“In Maqam 4, the place was calmer. I was sleeping better”.

“… but the only bad thing [sic] is that it has no sound insulation. Especially at weekends, you hear people sitting outside, someone walking in the corridor, or girls talking in adjacent rooms. This is its only defect”.

“The first thing is that our home is calm; so, the situation was suitable for me. In Maqam 4, we did not hear outside noise. It was calm. In the New Campus hostel, the walls are thin and so you can hear everything. The sound is very annoying, especially at night when we want to sleep. I preferred studying in the library than in my room”.

“The only negative about the NC hostel is that it is noisy. There is no insulation to block the noise from outside. It was very annoying. My room was in building 1, on the side of the emergency exit. So whenever someone opened or closed the door to this exit, I would wake up from my sleep. I am an engineering student, and our classes start at eight o’clock in the morning. So, it was very annoying … I hear you and your noise; how can I focus on my study?…Not all my classes are in person, and I have several online courses that are university requirements. With the noise outside and the teacher talking, I cannot concentrate”.

Another design element that was often mentioned by the respondents was represented by the distances to nearby facilities. This element mainly influenced the ease with which the students could access the facilities for satisfying their daily needs. For example, the respondents perceived as more convenient the shorter distances to the canteen and their classes from the NC hostel than from the Maqam 4 hostel.

“In the NC hostel, we are already within the university campus. If I want something, I can go back to the university to get it because it is close to me”.

“The only bad thing [sic] about the Maqam 4 hostel is that it is far from my classes, whereas the NC is close to the canteen and my classes. The buildings are very close to it, and so are the labs”.

The students also described their living experiences by indicating how they felt owing to some design-related elements of their dwellings. For example, they claimed that the presence/absence of sound insulation influenced their feeling of loneliness. While the Maqam 4 hostel was perceived by them to be very calm, some students felt scared and lonely. In the NC hostel, while some students had complained about noise, others construed the noise as a positive sign of life.

“Maqam 4 is very calm, to the extent that you feel lonely, really alone, while you don’t feel this in the NC hostel”.

Moreover, the students claimed that the use of dark versus light colors in the interior had a significant emotional impact on them. They described feeling depressed due to the use of brown colors in the corridors of Maqam 4, in contrast to the NC hostel, the white color of the walls of which lent a feeling of exhilaration to the students.

“But as a décor for the place, I felt, ‘no.’ Emotionally, Maqam 4 was hurting me a little. I felt that the place was closing in on me. The colors were green and purple. It was not helpful. It was nice in the NC building. I felt that the colors were lighter and exhilarating”.

“The only problem in Maqam 4 was that I felt the place was a little depressing because of the colors. The place is brown, and a bit scary”.

### 3.2. Students’ Perceptions of Their Emotional Well-Being

Table 1 shows the main design-related factors that the students claimed had an emotional influence on them. Their different feelings are described in correlation with various design-related aspects.

### 3.3. Students’ Perceptions of an Ideal University Hostel

Table 2 shows the students’ perceptions of the ideal hostel. They emphasized, in our interviews with them, the design-related factors that enhanced their emotional well-being in built environments.

## 4. Discussion

This study provided an in-depth analysis of students’ experiences and emotional well-being in their hostel living environments. The key takeaways stem from design-related aspects of the built environment, which significantly contribute to the emotional constructs of students. A primary factor emerging from the interviews was the role of acoustics, which students frequently associated with their emotional states. They identified correlations between the presence or absence of specific acoustic parameters and feelings of focus, privacy, comfort, annoyance, and fear. Therefore, acoustics are clearly influential in shaping the emotional well-being of students [8,9,10]. Equally vital are visual factors within the built environment, such as the color schemes used, the balance of artificial and natural lighting, and the views offered by the surroundings. Students associated these elements with a range of emotions including depression, comfort, motivation, energy levels, fear, and annoyance [13,15,18]. While private spaces, such as bedrooms, bathrooms, and study areas, were recognized for their contribution to privacy, students stressed the emotional impact of communal spaces. These spaces not only supported social interactions but also evoked feelings of comfort and excitement [21].

When envisioning their ideal hostel environment, students underscored the importance of nature exposure, both directly and indirectly. The students linked improved living experiences, involving more social interaction and focused studying, with the availability of a suitable outdoor landscape. This finding is supported by the literature on well-being in psychology [41,42]. The results also verified the importance of the support provided by multi-functional communal spaces for social interaction. This was identified as the main desideratum of the ideal hostel environment in our pilot study [21], and was also linked to nature. The absence of recreational and physical exercises has been associated with various mental health issues during COVID-19, such as stress, anxiety, and depression [43]. Multi-functional recreational and communal spaces can be used in the design of hostels that can be used in normal as well as extreme scenarios, such as COVID-19.

The physical design-related factors mentioned in this study, such as the size of the bedroom, the lighting, and surrounding views, have been found to influence students’ mental well-being in past work [13,15,18]. However, the feelings that the students expressed in our interviews can be related more intimately to their emotional well-being than to the presence/absence of certain mental concerns. Architecture often leans towards objective correlation between the built environment and emotions using biometric tools [8,9,10]. However, the subjective perspective rooted in the cognitive theory of emotions has been less examined. This study bridges this gap by using psychological interviews to elicit a diverse set of emotional constructs. Further research is necessary for their categorization [11]. It is important to mention that this study constituted an ambitious implementation of a recently considered aim concerning personal well-being and its relation to the built environment in the post-COVID-19 context, with a focus on individual differences in people’s sensitivity to experiences of built environments [22,23,44].

## 5. Conclusions

This qualitative study, to our knowledge, is the first to employ a psychological approach to examine students’ perceptions of their experiences within the built environment. The insights obtained highlight the distinct experiences students have in different built environments—their homes and university hostels. We were able to ascertain various feelings associated with certain design-related factors. By asking them to describe their ideal hostel, we gained insights into characteristics that amplify positive emotions and mitigate negative ones.

Acoustics, visual quality, privacy, exposure to nature, and multi-functional communal spaces are among the most common factors perceived by students to affect their emotional well-being. The emphasis of these factors appeared not only when students were asked about how the built environment influenced them emotionally, but they also mentioned the same design-related factors when they were asked to imagine an ideal university hostel to live in. Students perceived these design-related factors with relevance to a range of emotions, including depression, motivation, comfort, annoyance, fear, and loneliness. This perceived correlation between the design-related factors and emotions was described as bi-polar emotional constructs, such as feeling comfortable in a bedroom with good sound insulation versus feeling annoyed in a bedroom with bad sound insulation and feeling energetic with light interior colors versus feeling depressed with dark ones. Additionally, some design factors were perceived as promoting positive feelings in certain spaces and/or for certain purposes while having a negative influence in other cases. For example, students described a construct of feeling comfort with medium sound insulation in public spaces versus feeling lonely with optimum sound insulation in the same space. These elicited emotional constructs derived via the PCT and the qualitative RGT helped us to deeply understand students’ perceptions considering the studied context. More research is required to use these emotional constructs in a quantitative form of the RGT to study them for a larger sample. The study demonstrated the efficacy of the RGT, grounded in the PCT, in eliciting genuine student perceptions [25]. As opposed to speculative modeling in the design phase, this approach collects accurate experiential data that can greatly enhance post-occupancy evaluations. Through this understanding, we can more effectively identify factors shaping students’ experiences within these environments.

In the post-COVID-19 context, there is heightened awareness of the built environment’s influence on well-being, especially mental health. This sentiment is mirrored in numerous studies, with one notable example discussing an optimized responsive environment where various physical design aspects are significantly linked with integrative medicine practices, such as sleep, relationships, and movement [45]. The focus has shifted from merely mitigating negative impacts to actively enhancing positive ones, particularly concerning emotional well-being. This research endorses a more interactive, contextual approach to understanding users’ perceptions, moving beyond standardized scales [23]. However, the concept of emotional well-being within the built environment remains somewhat nebulous. With the World Health Organization’s consistent emphasis on mental well-being, as seen in their recent report “Transforming mental health for all” [46], it is clear that further research is needed. We must strive to understand people’s emotional needs better and create emotionally healthy built environments. This study contributes to this objective; however, there is more work to be carried out. Further research is crucial in developing a comprehensive theoretical framework addressing emotional well-being within the built environment. This work, in synergy with the previous discussion, highlights the significant emotional impacts of architectural design and underscores the necessity for increased focus in this area in future design practices.

## Figures and Tables

**Table 1:** Students’ perceptions of the main design-related factors influencing their emotional well-being.

Design-Related Factors	Common Perceptions
Availability/unavailability of private individual spaces	*“At home, I **like** studying at the desk, and this is available in the NC hostel; every student has her own desk in her room”.* *“Maqam 4 is better than the New Campus hostel in many ways. First, aspect there is **no sharing**”.* *“I feel that the hostel makes the student self-reliant. She is living in a room alone and must take care of herself. This is what **influences me positively**: I rely on myself. I have a room that I must clean and take care of organizing it. No one is taking care of it for me”.*
Sizes of spaces	*“In Maqam 4, it is wide, and this helps me cope with my **moods**. The NC hostel is narrower”.* *“The design of the room in Maqam 4 is **nice**. It is small, but you do not feel that it is too small. You feel that the bed and the cupboard are in appropriate places to make the room spacious. The space is not too **compacted**. This is not the case with the New Campus hostel”.* *“The New Campus has a small area. This does not allow space for easily rearranging the bed and the desk. You cannot put the desk near the pinboard as this means that the cabinet cannot be opened or takes up a lot of space. You cannot walk comfortably. This is the problem”*
Brightness/dimness of artificial lighting	*“The lighting. It was the weakest in the New Campus hostel. I am the kind of person where I want the room to be very well lit. I **feel good** in this case”.* *“In Maqam 4, the light was **not nice**. The place is **dull** and **obsolete** in the corridors, and everywhere else”.* *“The New Campus is more **comfortable;** even the lights are much **more comfortable**. I like the New Campus more than Maqam 4, where I did not like the white light in the bedroom. It was **annoying**. I feel that it would be better if it had lighting like that of the New Campus hostel”.*
Exposure to/restriction on natural views	*“I **loved** the gardens because I was studying outside a lot, and it had tables with chairs. I was in the appropriate **mood** studying there”.* *“The greenery outside is nice. It has a **nice impact**. When you open the window, you find lush views”.*
Lightness/darkness of interior colors	*“The colors in the Maqam 4 hostel are very dark. The rooms are pink, the curtains are pink, the table and the doors are brown. You understand. While the light is strong, the room is still **depressing**”.* *“The atmosphere is **depressing** because the colors in the hostel are generally dull. In the NC hostel, I feel a little more **energetic** than in Maqam 4 because of the colors”.* *“The NC hostel helps a lot because the colors in it are light, and this **motivates** us to study”.* *“The white color in the New Campus hostel is **motivating** and makes you feel **comfortable**. When I passed through the corridor, I did not feel **afraid** as I did in the corridors of Maqam 4, where I would run to reach my room because I found the brown color of the walls **scary**”.* *“What **annoys** me a little is the dark colors. For example, I told you about the pink walls and curtains in Maqam 4. It was not all one solid, dark color. No, it was decorated, but it was pink. It was **annoying**. The bathrooms in Maqam 4 were also a dark, burnt brown; so, it was a little annoying for me”.*
Openness/closedness of communal spaces	*“Sometimes, girls would bring an electric stove to the NC hostel and cook food. The rooms that are adjacent to the kitchen smell of food because the kitchen is not closed. The Maqam hostel has a kitchen with a door”.* *“Sometimes, I do not want to sit in the bedroom. I want to change, but the New Campus hostel has no study area. There is a place for sitting but I find that it is **not comfortable**. Girls are passing always”.*
Extent of natural light	*“The windows at home are very large; I **like** spaces with a lot of natural light”.* *“The windows in the New Campus hostel are large. This makes me **comfortable**. At home, my room has a lot of windows, and I like it when the sunlight enters my room. You know, you feel **energetic** in the morning. You got **excited** to do things and study”.*
Presence/absence of sound insulation	*“The insulation in Maqam 4 is very good. Even if I have a friend visiting, we do not hear the noise outside. We study and can **concentrate** in this space. While I was able to study in the NC hostel, the noise from outside interfered with the calm and **prevented me from concentrating** on studying. Maqam 4 is thus better for its environment for studying and **comfort**”.* *“There is no sound insulation in the New Campus. I feel that there is **no privacy**. Sometimes, when I talk to my family on the phone or with someone in my room, I need **privacy**. The girl in the adjacent room can hear me if she is in her bathroom or her bedroom. This is **annoying** for both of us. It is annoying for me because there are private things that I do not want someone to hear, and she can hear it. She is **annoyed** by having to hear my voice. This is not okay”.* *“Emotionally, I did not like Maqam 4 very much. It was very calm, to the extent that—I was online mostly—I felt that I was in a strange place that was **uncomfortable**. It was very calm; I could hear any sound. It was **scary**. This may be because at home, I am used to hearing my sisters’ voices, and other sounds. When I hear people around me, I am **more comfortable**. But Maqam is very calm”.*
Distances to nearby facilities	*“At home, it is the opposite: It is more **comfortable**. Everything is available; so, I do not waste time. Because the NC hostel is close to the canteen, I can go there and return quickly, and not **get tired**. When you walk from Maqam 4 to the canteen, you feel **tired** from walking. The NC is more **comfortable** in this sense”. * *“The proximity of the library to the NC hostel is **convenient**. When I want to get in the **mood** to study, I go to the library”.* *“Maqam 4 is a little far from the university and its colleges and is thus **scary**”.*
Supportive/unsupportive communal spaces for social interaction	*“In Maqam 4, I did not see many people. Everyone was mostly online, including all my friends, except me. I was mostly **alone**”.* *“I can spend more time with my friends in the NC hostel, unlike in Maqam 4. You also feel like you want to study. We all get **excited** and study”.* *“They moved me to the New Campus. The building is in a nice place. When we sit or walk outside, it is nicer and more **comfortable”.***

**Table 2:** Students’ perceptions of the main characteristics of an ideal hostel.

Main Characteristics	Common Perceptions
Availability of private spaces	*“I **like** the bedroom very much and do not see anything that needs to be changed. Especially, the idea of one bathroom for every two rooms is better than that of one bathroom for multiple rooms”.* *“Every student likes the NC hostel, and I **prefer** the allocation of rooms here. Everyone gets their own room, but the bathroom is shared. At least this provides **privacy** in the room. It is better to have a separate bathroom for each student, but if enough bathrooms are not available, it is fine to have to share it with someone I know. Not just anyone though”.*
Spacious sizes of spaces	*“It is a building where the rooms are not comfortable. The student should at least have the **comfort** of living in the room without feeling **squeezed**”.* *“The room is wide and there is space to put my stuff. You do not feel like you are in a narrow place and don’t get **worried**”.* *“For example, you can widen the bedrooms a little” + “For example, you can widen the corridors. I feel that they are very **narrow**”.*
Suitable artificial lighting	*“The lighting is **suitable**: not too **dim** and not too **strong**”.* *“The lighting **affects** me, especially at study time”.* *“The lighting should be **good**”.*
Exposure to natural views	*“There should be greenery inside the building, so that you **feel** the green space as you walk and smell the air and the plants”.* *“I feel the most important things are the greenery, plants, and the furniture. They lend the place more **beauty** and **comfort**”.* *“The gardens. Now that I am at home, I **miss** the gardens of the hostel”.* *“I feel the landscape outside is good for my **emotional well-being**, such that one wants to go outside”.* *“I feel like if it were up to me, I would make a wall of windows so I can see nature outside. In Maqam 4, the lounge has longitudinal windows so I can see outside. There is nature I can see. The only negative thing is that the windows of the lounges in Maqam 4 cannot be opened; so, there is no ventilation. I am one of the people who like studying at sunrise, but I cannot open the windows to **hear** the **birds** in the morning while I am studying, and to feel the fresh, cold air. So, I feel isolated. It needs ventilation”.* *“If you open the curtains, I think the entire **atmosphere** of the room changes”.* *“It would be **better** to have places for studying outside because there are chairs away from each other but not for studying. We usually bring tables from inside to outside and make a sitting area to study”.*
Use of light interior colors	*“The colors are **fresh** and include beige and white”.* *“Light colors should be used to make the students feel **comfortable** while they are studying”.* *“Regarding colors, I feel that it would be **nice** for the rooms to have some color: pastel colors on the walls”.*
Enclosure of certain communal spaces	*“When we want to study, we sit in any lounge space we find empty. But people passing by can see you studying, and not all of them are quiet. It is **nice** to have study rooms away from our bedrooms because the bedrooms themselves encourage sleeping. **Productivity** will increase if study rooms with coffee corners are made available. Something **exciting**”.* *“The living area in the New Campus is open; so, I **do not feel nice**. It is better to have a closed room so that the girls are **comfortable**. Moreover, there is no closed kitchen, so the smell of food from it reaches the rooms. It would be better if it was closed”.*
Presence of sound insulation	*“First, the place needs to have **privacy**. I mean no sound. Second, there should be a place that is private and calming so that the students can study, sleep, talk, be **comfortable** overall, and not be **annoyed**. Sometimes, I get **worried**. When I am taking an online class, I do not want to wear headphones but want to listen to the lecture without them, but I cannot do so because the girl in the adjacent room will be able to hear me and I do not want to bother her. I thus feel that sound insulation is very important”.* *“You should insulate the walls between the rooms. This is the most important thing to enable the student to **concentrate** on studying”.*
Availability of nearby basic facilities for daily needs	*“The place **motivates** the student and assures her that she is at home. For example, there is a pantry”.* *“The point is that the feeling you have is one of being **at home**, rather than in a university. You feel you are in your home and do not feel **strange,** or that you are in a faraway place. You have everything, like the kitchen and laundry. You do not have to go up and down or wait in a row. Everything is available”.* *“I want study rooms or study halls. The **atmosphere** in a hall is different from that in a room. This is the thing I have the biggest problem with”.* *“The study rooms are what I **miss** the most”.* *“The kitchen in our hostel now has nothing: only a microwave and a sink. We need to bring everything else, and when students cannot afford to do so, they cannot cook food and must eat the canteen food. It is nice to have a kitchen that is well **equipped** for students”.*
Availability of communal spaces encouraging social interaction	*“I feel they need to add study rooms, like in Maqam 4. There are study rooms there that **motivated** me and others to study. You are studying in a place where the other girls are studying as well”.* *“But it will be **nice** to have a gathering room for when we meet or watch TV or a movie”.*

**Figure A1:**
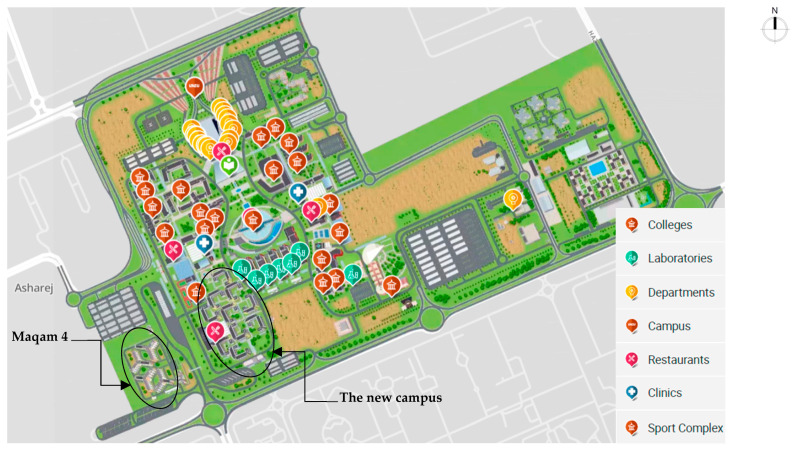
Locations of Maqam 4 and the New Campus hostels within the UAEU campus.

**Figure A2:**
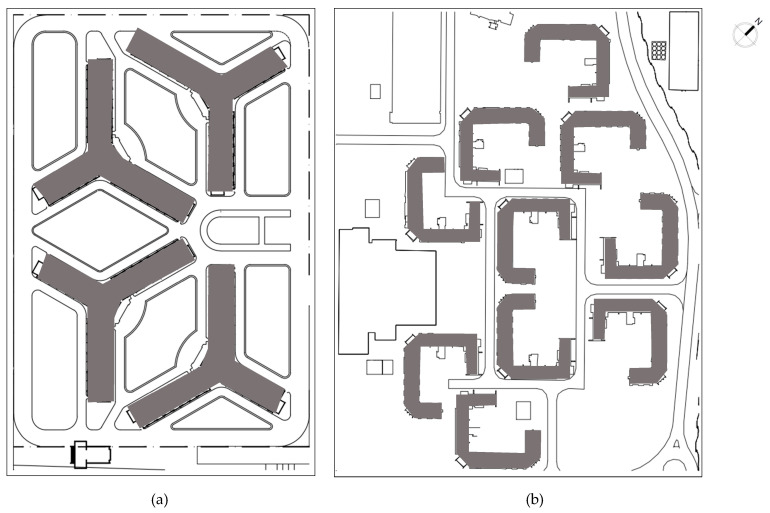
Site layout of the two hostels: (**a**) Maqam 4 and (**b**) New Campus.

**Figure A3:**
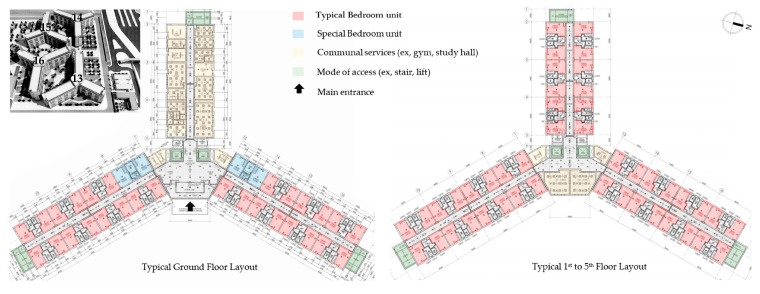
Typical ground and 1st to 5th floor layouts of Maqam 4 hostel, Building-16.

**Figure A4:**
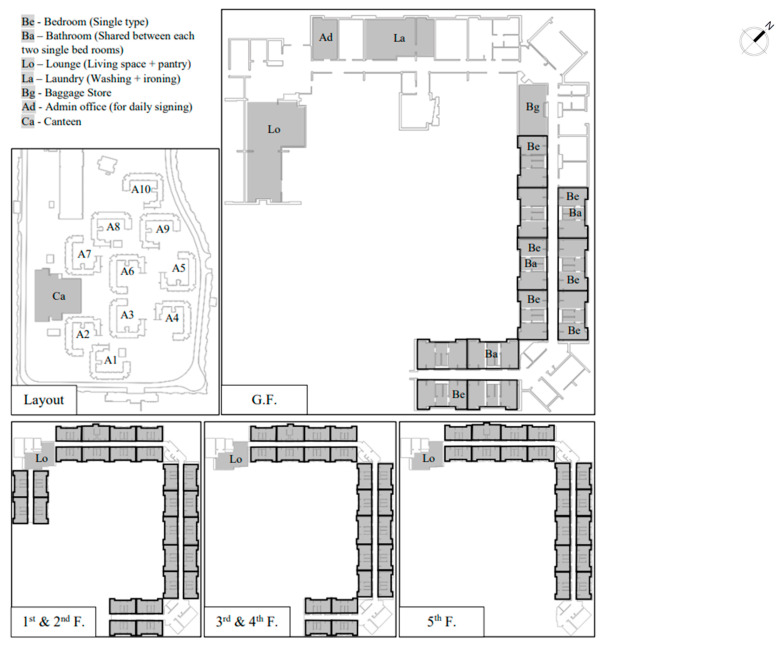
Typical ground and 1st to 5th floor layouts of the New Campus hostel, Building-A4.

**Figure A5:**
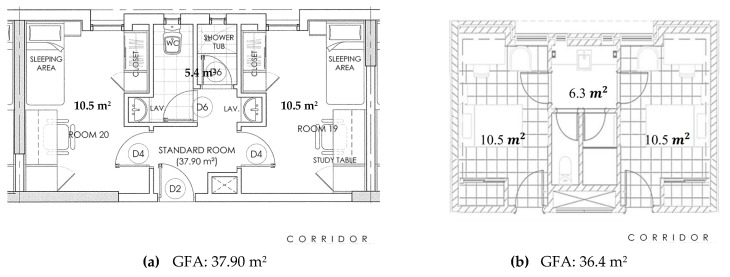
Typical standard room (1 student/room A or B): (**a**) Maqam 4 and (**b**) New Campus.

**Figure A6:**
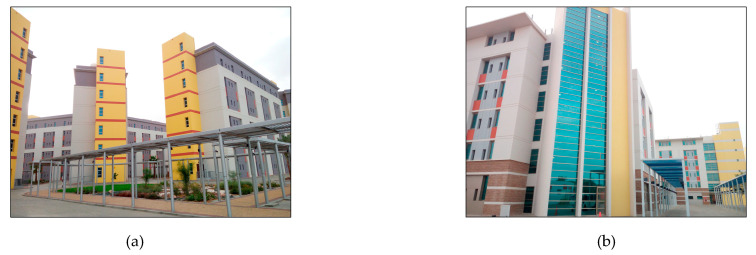
Exterior views of the two hostels: (**a**) Maqam 4 and (**b**) New Campus.

**Figure A7:**
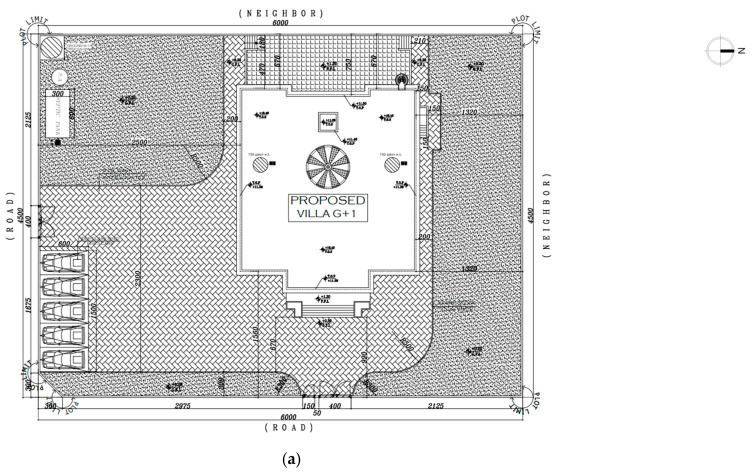

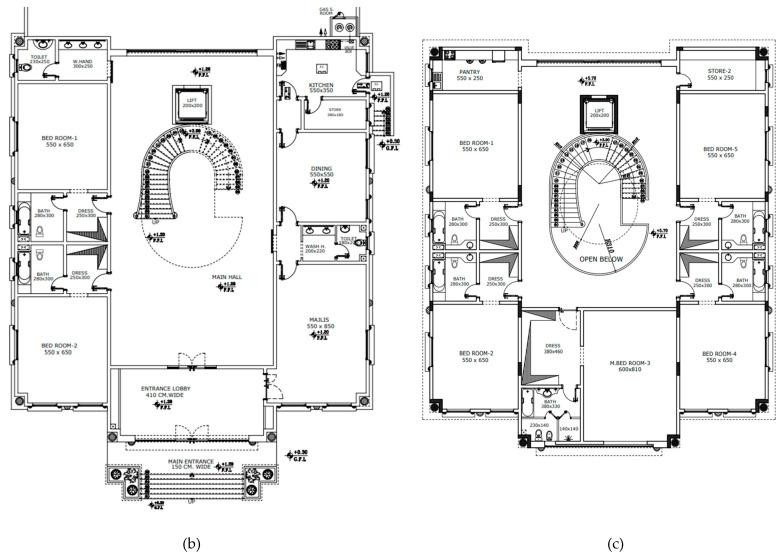
Case of an Emirati villa: (**a**) site layout, (**b**) G. F. plan, and (**c**) F. F. plan.

## Data Availability

The datasets generated or analyzed during this study are available from the corresponding author upon reasonable request.
